# FERREG: ferroptosis-based regulation of disease occurrence, progression and therapeutic response

**DOI:** 10.1093/bib/bbae223

**Published:** 2024-05-13

**Authors:** Yuan Zhou, Zhen Chen, Mengjie Yang, Fengyun Chen, Jiayi Yin, Yintao Zhang, Xuheng Zhou, Xiuna Sun, Ziheng Ni, Lu Chen, Qun Lv, Feng Zhu, Shuiping Liu

**Affiliations:** Key Laboratory of Elemene Class Anti-Cancer Chinese Medicines, Engineering Laboratory of Development and Application of Traditional Chinese Medicines, Collaborative Innovation Center of Traditional Chinese Medicines of Zhejiang Province, School of Pharmacy, and Department of Respiratory Medicine of Affiliated Hospital, Hangzhou Normal University, Hangzhou, 311121, China; College of Pharmaceutical Sciences, The Second Affiliated Hospital, Zhejiang University School of Medicine, Zhejiang University, Hangzhou, 310058, China; Key Laboratory of Elemene Class Anti-Cancer Chinese Medicines, Engineering Laboratory of Development and Application of Traditional Chinese Medicines, Collaborative Innovation Center of Traditional Chinese Medicines of Zhejiang Province, School of Pharmacy, and Department of Respiratory Medicine of Affiliated Hospital, Hangzhou Normal University, Hangzhou, 311121, China; Key Laboratory of Elemene Class Anti-Cancer Chinese Medicines, Engineering Laboratory of Development and Application of Traditional Chinese Medicines, Collaborative Innovation Center of Traditional Chinese Medicines of Zhejiang Province, School of Pharmacy, and Department of Respiratory Medicine of Affiliated Hospital, Hangzhou Normal University, Hangzhou, 311121, China; Department of Clinical Pharmacy, The First Affiliated Hospital, Zhejiang University School of Medicine; College of Pharmaceutical Sciences, The Second Affiliated Hospital, Zhejiang University School of Medicine, Zhejiang University, Hangzhou, 310058, China; College of Pharmaceutical Sciences, The Second Affiliated Hospital, Zhejiang University School of Medicine, Zhejiang University, Hangzhou, 310058, China; College of Pharmaceutical Sciences, The Second Affiliated Hospital, Zhejiang University School of Medicine, Zhejiang University, Hangzhou, 310058, China; Key Laboratory of Elemene Class Anti-Cancer Chinese Medicines, Engineering Laboratory of Development and Application of Traditional Chinese Medicines, Collaborative Innovation Center of Traditional Chinese Medicines of Zhejiang Province, School of Pharmacy, and Department of Respiratory Medicine of Affiliated Hospital, Hangzhou Normal University, Hangzhou, 311121, China; Key Laboratory of Elemene Class Anti-Cancer Chinese Medicines, Engineering Laboratory of Development and Application of Traditional Chinese Medicines, Collaborative Innovation Center of Traditional Chinese Medicines of Zhejiang Province, School of Pharmacy, and Department of Respiratory Medicine of Affiliated Hospital, Hangzhou Normal University, Hangzhou, 311121, China; Department of Respiratory, The Affiliated Hospital of Hangzhou Normal University, Hangzhou, 311121, China; College of Pharmaceutical Sciences, The Second Affiliated Hospital, Zhejiang University School of Medicine, Zhejiang University, Hangzhou, 310058, China; Innovation Institute for Artificial Intelligence in Medicine of Zhejiang University, Alibaba-Zhejiang University Joint Research Center of Future Digital Healthcare, Hangzhou, 330110, China; Key Laboratory of Elemene Class Anti-Cancer Chinese Medicines, Engineering Laboratory of Development and Application of Traditional Chinese Medicines, Collaborative Innovation Center of Traditional Chinese Medicines of Zhejiang Province, School of Pharmacy, and Department of Respiratory Medicine of Affiliated Hospital, Hangzhou Normal University, Hangzhou, 311121, China

**Keywords:** ferroptosis, disease progression, drug response, cancer

## Abstract

Ferroptosis is a non-apoptotic, iron-dependent regulatory form of cell death characterized by the accumulation of intracellular reactive oxygen species. In recent years, a large and growing body of literature has investigated ferroptosis. Since ferroptosis is associated with various physiological activities and regulated by a variety of cellular metabolism and mitochondrial activity, ferroptosis has been closely related to the occurrence and development of many diseases, including cancer, aging, neurodegenerative diseases, ischemia–reperfusion injury and other pathological cell death. The regulation of ferroptosis mainly focuses on three pathways: system Xc^−^/GPX4 axis, lipid peroxidation and iron metabolism. The genes involved in these processes were divided into driver, suppressor and marker. Importantly, small molecules or drugs that mediate the expression of these genes are often good treatments in the clinic. Herein, a newly developed database, named ‘FERREG’, is documented to (i) providing the data of ferroptosis-related regulation of diseases occurrence, progression and drug response; (ii) explicitly describing the molecular mechanisms underlying each regulation; and (iii) fully referencing the collected data by cross-linking them to available databases. Collectively, FERREG contains 51 targets, 718 regulators, 445 ferroptosis-related drugs and 158 ferroptosis-related disease responses. FERREG can be accessed at https://idrblab.org/ferreg/.

## INTRODUCTION

Ferroptosis is a form of regulated cell death that occurs due to lipid peroxidation resulting from the accumulation of reactive oxygen species (ROS) generated by iron overload, leading to damage to the cell membrane [1, 2]. Extensive publications have revealed the target genes involved in the three metabolic pathways of ferroptosis: system Xc^−^/GPX4 pathway, lipid metabolism pathway and iron metabolism pathway [3–5]. Ferroptosis exhibits a momentous role in the onset and progression of diverse diseases [6]. For instance, ferroptosis can effectively kill tumor cells in colorectal cancer, thus inhibiting the malignant progression of cancer [7]. Conversely, the occurrence of ferroptosis results in damage or functional loss in normal cells during ischemia–reperfusion injury [8] and liver disease conditions [9]. Exploring the potential impact of ferroptosis on disease development and drug response has emerged as a key research focus in recent years [10–13]. Several studies have unveiled the molecular mechanisms that activated or inhibited ferroptosis, consequently influencing the occurrence and progression of various diseases [14–17].

So far, only two ferroptosis-related databases, FerrDb [18] and ncFO [19], have been developed. FerrDb focused on describing ferroptosis regulators, markers and associated diseases. ncFO, in particular, paid attention to the impact of non-coding RNAs (ncRNAs) on ferroptosis. Both databases have obtained significant attention and are widely cited. However, to the best of our knowledge, there is currently no available database that comprehensively provides data on the regulation of ferroptosis in disease occurrence, progression and drug response. There is an urgent need for a specific and systematic description of the molecular mechanisms of each regulator/compound in the regulation of ferroptosis target genes.

Herein, a database called ‘FERREG: ferroptosis-based regulation of disease occurrence, progression and therapeutic response’ was constructed. First, a systematic literature review was conducted on PubMed to obtain the ferroptosis alterations related to the occurrence and development of various diseases and the response data of ferroptosis. Second, the targets involved in the ferroptosis were carefully identified and categorized into driver, suppressor and marker. The regulators or drugs regulating ferroptosis targets were identified and categorized. Finally, all the data in FERREG have been fully cross-linked to diverse well-distinguished ferroptosis-related databases [20–25]. Comprehensive information on the interplays between targets, regulators and drugs, as well as data on the changes of disease occurrence, progression and therapeutic response, was provided in FERREG. It is believed that FERREG will have a significant impact on drug development and disease treatment strategies related to ferroptosis. FERREG is now free and open to all users without login requirement at: https://idrblab.org/ferreg/.

## RESULTS

### The ferroptosis target genes and their biological function

**Figure 1:**
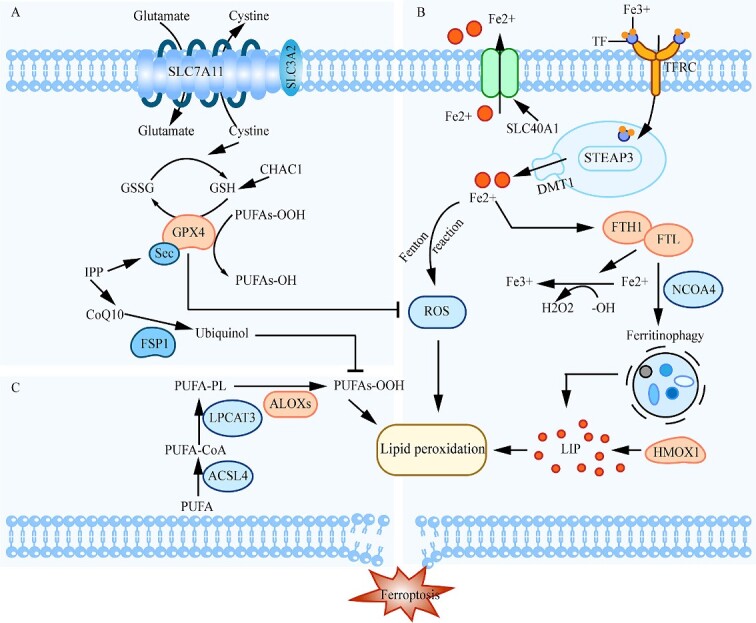
Metabolic pathways of classical ferroptosis. (**A**) The cystine/glutamate antiporter (system Xc^−^), composed of SLC7A11 and SLC3A2, is responsible for the intracellular import and conversion of cystine to cysteine for the synthesis of glutathione (GSH). The glutathione peroxidase 4 (GPX4) can reduce the endogenous neutralization of PUFAs-OOH to PUFAs-OH and ultimately reduce the accumulation of ROS. (**B**) Extracellular Fe^3+^ binds to transferrin (TF) and enters the cell through its receptor (TFRC). Subsequently, Fe^3+^ is reduced to Fe^2+^ by six-transmembrane epithelial antigen of prostate 3 (STEAP3), It is transported to the cytoplasm by divalent metal transporter 1 (DMT1) to form labile iron pool (LIP). In addition, FTH1/FTL, the ferritin component, also increases LIP by participating in ferroptosis, which leads to cell ferroptosis. (**C**) The important link of ferroptosis is the dysregulation of iron-dependent lipid metabolism. The long-chain-fatty-acid-CoA ligase 4 (ACSL4) catalyzes the binding of free arachidonic acid (AA) or epinephrine acid (AdA) to coenzyme A to form derivative PUFA-CoA, which is lysophosphatidylcholine acyltransferase 3 (LPCAT3) is esterified to PUFA-PL and oxidized by lipoxygenase (ALOXs) to form lipid peroxidation products, which induce ferroptosis.

The characteristics of ferroptosis can be attributed to three basic elements [26–28]: substrate of lipid peroxidation (generated by ACSL4, LPCAT3, etc.), iron-mediated oxidative damage (facilitated by SLC11A2, NOX1, etc.) and the anti-ferroptosis system (functioned by GPX4, SLC7A11, etc.). The mechanism diagram of how the three regulate ferroptosis is shown in Figure 1. The targets associated with these three metabolic pathways can be classified as driver, suppressor and marker, and any molecular changes or drug interventions may affect the ultimate consequences of ferroptosis [29].

#### The biological function of drivers, suppressors and markers

During ferroptosis, polyunsaturated fatty acids (PUFAs) are most susceptible to peroxidation, which can cause the destruction of the lipid bilayer and affect membrane function. ACSL4 could act as a ferroptosis driver via catalyzing the linkage PUFAs to coenzyme A (CoA) and participating in the process of lipid peroxidation, which requires the involvement of ROS [30–32]. Excessive ROS is produced in an iron-dependent manner. There are several facets that would influence the iron level and have an impact on ferroptosis. The serum transferrin receptor (TFRC) complex loaded with iron is internalized in the endosomes, where they release iron (Fe^2+^) into the cytoplasm through SLC11A2 [33]. On the contrary, ferritin is an iron storage protein complex, comprising FTH1 and FTL, that prevents Fe^2+^ from being oxidized by ROS [34–36]. The antioxidant enzyme GPX4 can directly reduce ROS, thus acting as a central suppressor of ferroptosis in cancer cells [37]. The effective antioxidant effect of GPX4 relies on glutathione (GSH) as a coenzyme factor, which requires system Xc^−^ (consisting of two subunits, SLC7A11 and SLC3A2) to be able to import cysteine into cells for subsequent GCL-mediated GSH production [38]. In addition, certain genes and proteins, such as PTGS2 [39], CHAC1 [40] and TFRC [41], have been characterized as ferroptosis markers in preclinical models. The changes in their expression levels indicate the occurrence or termination of ferroptosis.

Changes in target genes related to ferroptosis can have a significant impact on the disease occurrence, progression and therapeutic response. Take FTH1 as an example, the downregulation of circSnx12 and the upregulation of miR-224-5p could lead to the downregulation of FTH1, directly regulating iron overload in myocardial cells and ultimately leading to cardiac cell death [42]. Another example involved the indirect regulation of FTH1 by the natural drug curcumenol to trigger ferroptosis in lung cancer, thereby inhibiting the proliferation of lung cancer cells. Mechanistically, curcumenol can induce the downregulation of the lncRNA H19, reduce its binding to miR-19b-3p, significantly increase the expression of miR-19b-3p and attenuate the transcriptional activity of FTH1, thereby inducing ferroptosis [43]. Additionally, loss of GPX4 function resulted in lapatinib-resistant breast cancer cell ferroptosis *in vitro* and prevented tumor relapse *in vivo* [44]. All these findings highlighted the potential of targeting ferroptosis-related genes in cancer and injury-associated diseases, offering new opportunities for diagnosis and therapeutic interventions [45].

#### The description and statistics of target genes in FERREG

**Figure 2:**
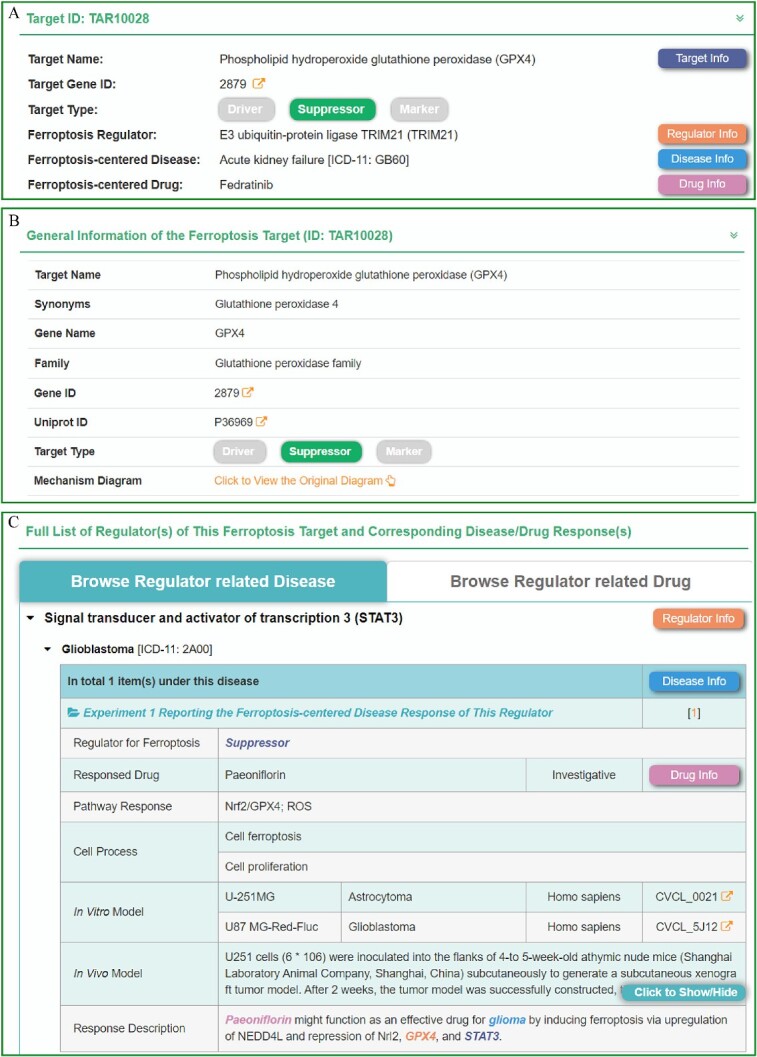
A typical FERREG webpage for target. (**A**) Take ‘GPX4’ as an example; search for each target will show relevant regulator, disease, drug and corresponding buttons with different colors. (**B**) Target’s detail page and the presentation of the mechanism diagram. (**C**) The specific mechanisms regulating ferroptosis target genes in different diseases.

On the ‘Home’ page of the FERREG database, users could easily locate the ‘Target’ column, where they could click to search and browse the related ferroptosis genes freely. Users could access relevant target information via the target name directly or based on their specific interests, including regulators, specified diseases and therapeutic drugs. A typical webpage for a target (GPX4) in FERREG is illustrated in Figure 2. The webpage provided comprehensive information about the target, including its name, synonyms, gene name, sequence, family, biological function, target type (driver, suppressor or marker) and external links to other well-known databases [46–52]. Additionally, FERREG offered a panoramic diagram illustrating the regulation of each target by regulators and drugs, as well as a bar graph depicting its expression patterns in 32 tissues.

In addition to providing basic information about the target, FERREG offered a comprehensive list of experimentally validated disease progression and drug reactions related to the studied target. All regulatory information was initially classified based on the regulator and further categorized according to the associated disease or drug, which facilitated users to choose the browsing mode of their interests. The detailed regulatory information, such as the role of regulator for ferroptosis, responded drug, related pathway, cell process and *in vitro*/*vivo* model, were systematically provided in FERREG.

As shown in Table 1, the FERREG database covered 51 ferroptosis targets (34 drivers, 26 suppressors and 8 markers) engaged in the three metabolic pathways, with 16 genes classified into two/three categories simultaneously. All these targets were mediated by 718 regulators, including 471 protein coding genes, 130 miRNA, 55 lncRNA, 47 circRNA, 14 precursor RNA and 1 pseudogene. Additionally, comprehensive information on their mutual regulation, along with important excerpts from relevant literature, was also provided. Mechanistic studies have revealed that there were 32 signaling pathways (such as PI3K/AKT pathway, autophagy and necroptosis) that regulated ferroptosis in 158 human diseases. Moreover, a comprehensive collection of 445 drugs with clinical therapeutic importance has been included, which directly or indirectly affect the target genes, leading to the induction or suppression of ferroptosis.

**Table 1:** A summary of targets, regulators, drugs and diseases for FERREG

Type	Count
Target^a^	
Driver	34
Suppressor	26
Marker	8
Regulator	
Protein	471
miRNA	130
lncRNA	55
circRNA	47
Precursor RNA	14
Pseudogene	1
Drug	
Small molecule	423
Protein	4
Others	18
Disease	
Oncology	47
Neurology	15
Digestive	12
Cardiovascular	11
Others	73

^a^16 genes classified into two/three categories simultaneously.

### The ferroptosis-centered regulation of disease development

As a nascent field, an increasing body of research demonstrated the pivotal role of ferroptosis in a wide range of pathological processes and diseases [53–57]. It has been revealed that the lactate inhibited the ferroptosis of liver cancer cells through the HCAR1/MCT1-SREBP1-SCD1 pathway, which potentially contributes to tumor metastasis and development [58]. What’s more, diabetes has been shown to aggravate myocardial ischemia–reperfusion injury in a ferroptosis induction manner [59–61]. Ferroptosis has become increasingly recognized as an important process that mediates the pathogenesis and progression of numerous cardiovascular diseases, including atherosclerosis, drug-induced heart failure and myocardial ischemia–reperfusion injury.

**Figure 3:**
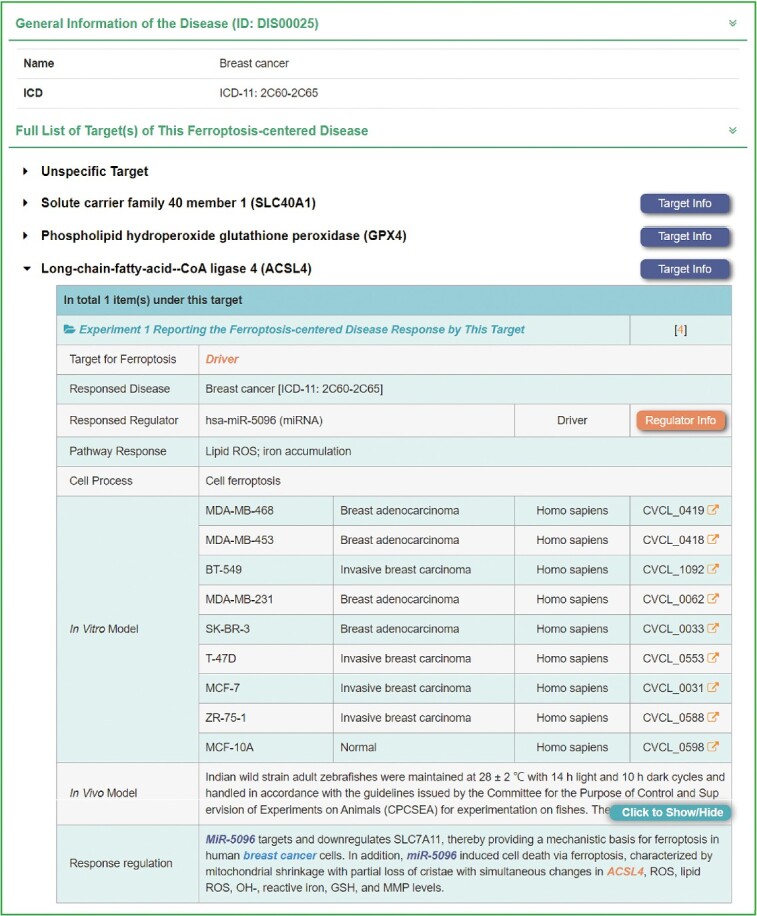
A typical FERREG webpage for disease. On the ‘Disease’ page, users can search directly by disease name or by disease-related ferroptosis target genes or regulators. Taking ‘Breast cancer’ as an example, this review presents the detailed mechanism of ferroptosis-centered disease response, including the involved ferroptosis-related target genes and regulators and their regulatory relationships, response pathways, cellular processes, *in vitro* and *in vivo* models and a summary of the disease response.

Therefore, an interface for searching diseases has been developed in FERREG, where users can manually input the disease name or select the disease of interest via a dropdown list. All ferroptosis-related diseases were standardized according to the latest International Classification of Disease [62], and a total of 158 diseases were identified to be closely related to ferroptosis. As shown in Figure 3, the disease development information provided in FERREG included: (a) The targets and regulators involved in this disease, while providing the ‘Target Info’ button and the ‘Regulator Info’ button links to the detailed information page respectively. (b) The cancer-related metabolic pathways involved in disease progression, such as autophagy, glycolysis, m^6^A methylation and Hippo/Yap signaling pathway. These metabolic pathways enable users to gain a better understanding of the specific processes and molecular mechanisms of ferroptosis in various diseases. (c) Numerous cellular processes involved in each experimental entry of the disease response, such as cell apoptosis, cell autophagy and cell necroptosis. Additionally, processes like cell proliferation, cell migration and cell invasion, which are closely related to disease progression, are also included. (d) The *in vitro* and *in vivo* models were used to investigate the regulation of ferroptosis. A total of 802 cell lines from different diseases were included in the FERREG database. (e) An elaborated description on the molecular mechanism mentioned in the literature, entitled ‘Response regulation’ in FERREG. The target, regulator, disease and drug were annotated in color to facilitate users’ understanding of the interplay among them in diseases.

### The ferroptosis-centered regulation of drug development

The interaction between targets and regulators alters disease development by influencing ferroptosis and increasing the complexity of the disease. However, in other words, potential therapeutic targets can be identified by elucidating the molecular mechanisms related to ferroptosis [63–65]. The identification of ferroptosis target genes (such as GPX4, FSP1 and TFRC) and multiple regulators (such as ZFAS1 and NEAT1) has provided novel opportunities for drug design [66]. Taking flubendazole as an example, the FDA-approved anthelmintic drug has been found to downregulate the expression of SLC7A11 and GPX4 in a targeted manner to p53, activating ferroptosis and exerting the anti-proliferative and pro-apoptotic effects in castration-resistant prostate cancer [67]. The manipulation of ferroptosis through drug-target/regulator interactions holds promising clinical prospects for disease treatment or enhancing the sensitivity of chemotherapy drugs [68].

**Figure 4:**
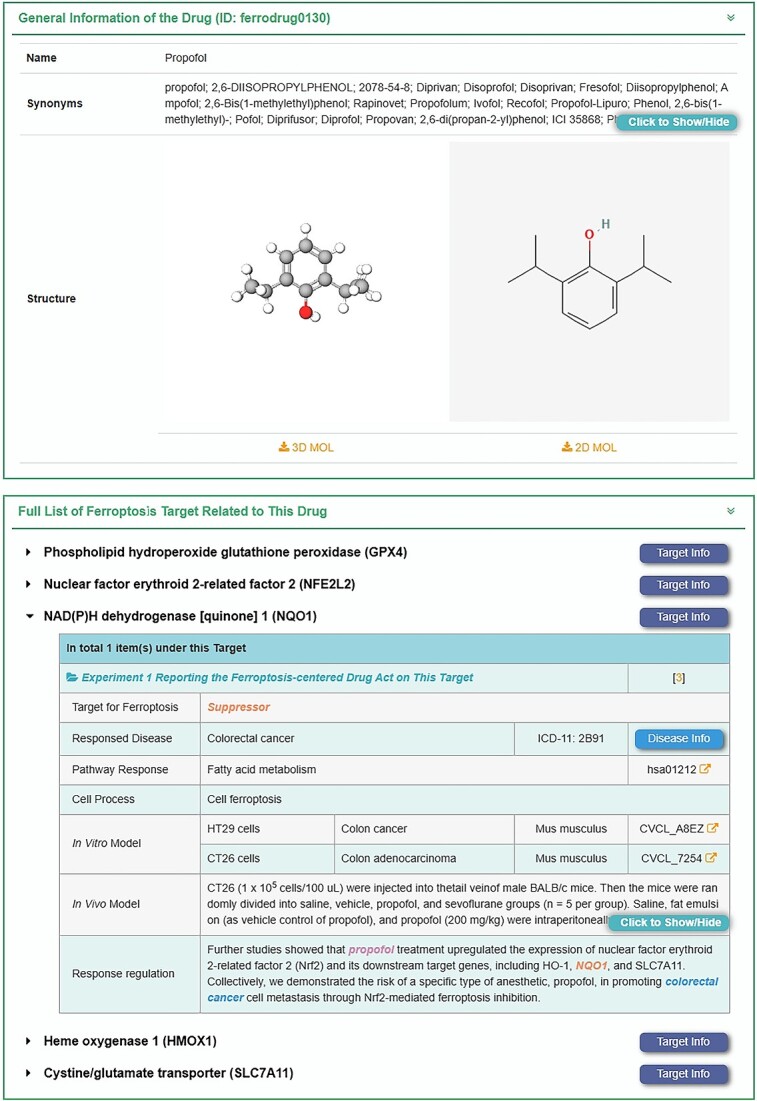
A typical FERREG webpage for drug. The ferroptosis-centered drug response is demonstrated using ‘Propofol’ as an example. The basic information of the drug is first presented, followed by a clear mechanism description for the different target genes of the drug. Regulatory information includes associated diseases, regulatory pathways and experimentally validated *in vitro* and *in vivo* models. The last column is for detailed regulatory information, and the key points mentioned are colored.

The webpage for the drug response in the FERREG database is shown in Figure 4. This page presented a ‘Ferroptosis-centered Drug Response’ table, which included 445 drugs that selectively influenced ferroptosis-related pathways and altered the occurrence and development of diseases. Users could find drugs based on the drug name, associated targets/regulators or the diseases. The retrieved results provided general information about the drugs, including drug name, synonyms, clinical status, drug type, structure, formula, International Union of Pure and Applied Chemistry (IUPAC) name, canonical SMILES, InChI, InChIKey and IDs sourced from various databases such as PubChem and Therapeutic Target Database (TTD). Furthermore, detailed information regarding drug regulation of ferroptosis is provided, including drug–target interactions, response diseases, pathway responses, cellular processes, validation through *in vitro* and *in vivo* models and the underlying mechanisms of drug regulation. Importantly, references to the original literature are provided in the bottom bar of all interfaces for easy access. Advancements in ferroptosis research have led to the discovery of a series of approved drugs (e.g. sulfasalazine [69], artesunate [70], sorafenib [71], acetaminophen [72]) that could induce ferroptosis. Moreover, there is an increasing emphasis on utilizing ferroptosis targets and regulators as the basis for large-scale screening of small-molecule drugs or developing larger-molecule drugs, such as specific antibodies. It is anticipated that the drug response data related to ferroptosis provided by FERREG can offer valuable insights for the development of drugs targeting ferroptosis [73].

## DISCUSSION

Ferroptosis is a form of programmed cell death induced by iron-dependent lipid peroxidation, which is the result of the confrontation between its oxidation and antioxidant systems [74]. Extracellular Fe^3+^- transferrin complex forms an endosome through TFRC and enters the cell, which is reduced by STEAP3 and transported to the cell by DMT1 in Fe^2+^ form. Both ROS and LIP, derived from Fe^2+^, can peroxidize PUFAs produced by ACSL4 and LPCAT3, then initiating ferroptosis. The antioxidant system composed of Xc^−^/GPX4 can reduce the peroxidation, thereby inhibiting ferroptosis. Currently, numerous regulators play a significant role in influencing key components of the oxidative/antioxidant system, either activating or inhibiting the occurrence of ferroptosis. Clarifying the regulation of regulators on targets and their impact on ferroptosis is crucial for understanding the mechanisms of disease development [75–77].

Since its introduction in 2012, ferroptosis has offered a novel perspective for investigating chronic diseases characterized by intricate pathological mechanisms [3]. Exploring the potential mechanisms of ferroptosis in prevalent chronic conditions such as oncology [78], neurodegenerative disorders [79], cardiovascular [80] and digestive system diseases [81] can provide a fresh foundation for their prevention and management. It is important to strictly confirm the role of ferroptosis in biological processes in diseases [82]. Recent findings have highlighted the interplay between ferroptosis and other forms of cell death, particularly apoptosis and autophagy characterized by autolysosomes [83–85]. Studies have found that the activation of autophagy can degrade ferritin and then induce ferroptosis in pancreatic ductal adenocarcinoma/fibrosarcoma cells [86–88].

Further research on the molecular mechanism of ferroptosis has broadened the potential therapeutic intervention. Numerous studies have confirmed that eliminating tumor cells through ferroptosis can prevent drug resistance and enhance drug sensitivity [89]. Inducers and inhibitors of ferroptosis have shown promising therapeutic effects within a variety of diseases [90–92].

**Figure 5:**
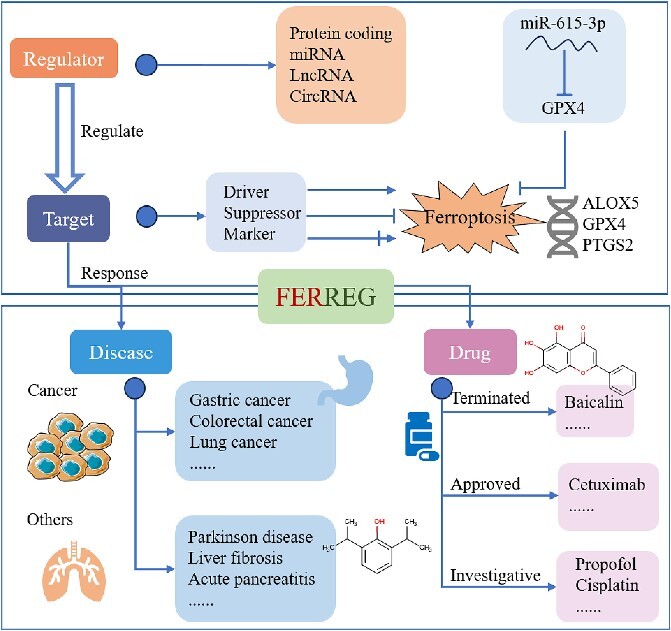
Ferroptosis targets and regulators involved in regulating disease and drug response were collected in FERREG. The target genes in the ferroptosis signaling pathway are classified as ‘Driver’, ‘Suppressor’ and ‘Marker’. Ferroptosis regulators can promote or inhibit the occurrence of ferroptosis by regulating these targets.

As shown in Figure 5, the FERREG database has been developed as a valuable resource for researchers, offering a series of disease development and drug response databases focused on experimentally validated ferroptosis regulatory genes. Our database highlights how ferroptosis is regulated by various regulators and how these regulators are associated with disease progression and drug response. The inclusion of the mechanism diagrams and specific examples makes our FERREG convenient for users to conduct multi-level and multi-dimensional correlation analysis between ferroptosis, diseases and drugs. As an increasing number of scientists focus their studies on ferroptosis, a large and growing body of literature will emerge to reveal the mechanisms of ferroptosis in disease occurrence, progression and drug response. We will therefore constantly update the FERREG database during the coming decade.

## MATERIALS AND METHODS

### Data collection for ferroptosis-centered regulation

In order to collect the data on ferroptosis-centered regulation of disease occurrence, progression and therapeutic response, a comprehensive literature review related to ferroptosis was conducted in the PubMed database using a series of keywords: ‘ferroptosis + target gene name’, ‘ferroptosis + drug’, ‘ferroptosis + disease’, ‘ferroptosis target gene name+ drug’, ‘ferroptosis target gene name + disease’, ‘ferroptosis + regulator’, ‘regulator name + drug’, ‘regulator name + disease’, etc. A total of 51 ferroptosis target genes (such as GPX4, ACSL4 and SLC7A11) and 718 ferroptosis regulators (such as SIRT3, FBXW7 and H19), which could regulate the expression and function of targets, under 158 disease conditions (such as ischemia/reperfusion injury, acute myeloid leukemia, hereditary leiomyomatosis) were collected in FERREG. Furthermore, the response data of 445 drugs (such as baicalein) that would induce or inhibit ferroptosis under pathological conditions and have clinical significance were included. For these ferroptosis targets and regulators, essential gene features were collected from TTD [93, 94], UniProt [23], KEGG [24] and miRbase [95] databases. Finally, each publication was carefully reviewed, and a list was compiled with detailed information including regulatory information, response pathways, cell processes, tissues/cell lines, *in vivo* models and so on.

### FERREG data standardization, access and retrieval

To facilitate user utilization and maintain data consistency, all raw data in FERREG have been normalized. Data standardization includes the following three aspects: first, the target genes and regulators in this website are named based on well-known databases like NCBI Gene, UniProt and miRbase. Users can conveniently access the corresponding database by clicking on a provided link. Secondly, all diseases in FERREG have been standardized according to the latest version of the International Classification of Diseases. Thirdly, the database offers external links to drug resources such as PubChem, TTD and DrugBank. These links allow users to access more detailed information about drugs or compounds online, eliminating the need for additional logins. These measures have been implemented to enhance user experience and ensure the availability of comprehensive and accurate information within the database.

**Key Points**

- The FERREG provides data on ferroptosis-related regulation of disease occurrence, progression and drug response.
- The FERREG explicitly describes the molecular mechanism underlying each regulation.
- The FERREG fully references the collected data by cross-linking them to well-established databases.

## DATA AVAILABILITY

All the ferroptosis-relevant data could be viewed, accessed and downloaded from FERREG, which could be freely accessed without any login requirement at https://idrblab.org/ferreg/.

## Author Biographies

**Jiayi Yin** is a special associate researcher of Department of Clinical Pharmacy, The First Affiliated Hospital, Zhejiang University School of Medicine. She specializes in precision medical research.

**Zhen Chen**, **Yintao Zhang**, **Xiuna Sun** and **Xuheng Zhou** are students in the College of Pharmaceutical Sciences of School of Pharmaceutical Sciences in Zhejiang University, Hangzhou 310058, China. They are interested in bioinformatics and molecular biology.

**Yuan Zhou**, **Ziheng Ni**, **Mengjie Yang**, **Fengyun Chen** and **Lu Chen** are students of School of Pharmaceutical Sciences in Hangzhou Normal University, Hangzhou 310058, China. They are interested in bioinformatics and molecular biology.

**Qun Lv** is a doctor of Department of Respiratory Medicine in Affiliated Hospital, Hangzhou Normal University, Hangzhou 310058, China. His current focus includes bioinformatics.

**Feng Zhu** is a Tenured Full Professor of College of Pharmaceutical Sciences in Zhejiang University, Hangzhou 310058, China. His research laboratory (https://idrblab.org/) has been working in the fields of bioinformatics, OMIC-based drug discovery, system biology and medicinal chemistry.

**Shuiping Liu** is an associate professor and master's supervisor at Hangzhou Normal University, Hangzhou 310058, China. His research focuses on the mechanism of epigenetic modifications such as DNA methylation and m6A methylation in cancer.
